# Varieties in Disease

**Published:** 1858-04

**Authors:** Thomas Pollard

**Affiliations:** Richmond, Virginia


A T L A N T A

### Medical and Surgical Journal.

Vol. III.] APRIL, 1858. [No. VIII.

### ORIGINAL COMMUNICATIONS.

### ARTICLE 1.

*Varieties in Disease.* By Thomas Pollard, M. D., Richmond, Virginia.

Every old Practitioner must have been struck with the difference between disease, as described in the books, and as found at the bed-side, and we consider it our duty to warn the young Practitioner, and Medical student of this difference. The descriptions of disease as found in the standard authors is in the main, no doubt correct, but their limited space, and probably want of sufficient pains with some of them, have precluded the mention of many of the irregularities of morbid action.

We propose in a cursory manner, to notice a few of these varieties, enough to show that we must observe for ourselves, and not take the dicta of Medical writers as our guide.

One of the diseases exhibiting many varieties is "Scarlet Fever." We do not always find it coming out on the second or third day of the fever. In some severe cases it does not come out at all, debility, and want of reaction, and finally death preventing it. The writer has seen a case where the eruption was the first symptom, followed 24 hours after by the Fever. In this case the eruption appeared first on the arms and breast, and the face, stopping for ten hours by well defined limits, like Erysipelas, on the middle of the arms and chest, and then travelling over the remainder of the body by degrees This case was well marked, and other cases occurred in the family, one of which terminated fatally, thus proving there could be no mistake in the diagnosis. It is said the eruption of Scarlet Fever is not raised above the surface, but in some cases it is, occasionally assuming a vesicular form, the vesicles resembling sudamina, and then again it is sometimes pustular. The books describing the eruption say it remains out 3 or 4 days, and then begins to decline; whereas, it sometimes remains out 3 or 4 weeks.

Another disease presenting many phases in its symptoms is Typhoid Fever. The popular belief, in which many Medical men seem to participate, is that the affection is necessarily severe, the fact being as we believe, that like most other diseases, it presents all the diversities, from the mildest to the severest form—from malaise, frequent pulse, and languor with ability to keep about, with, or without a little irritation about the bowels, to the well marked fever with the pulse mounting to 150 or 160, Diarrhœa, disturbance about the brain, with delirium, bronchial complication, red fiery tongue, sordes on the gums and mouth, and all the concomitants which go to make up a fearful malady, in fully formed Typhoid Fever. It may be asked how these mild cases in patients scarcely sick enough to go to bed, are recognised. Principally by the attending facts and circumstances, and by exclusion of other diseases from consideration, which have none of these symptoms. Where other cases of the well marked disease are occurring in the family, and these mild attacks supervene in other members, and cannot be attributed to any thing else; we must conclude they are Typhoid Fever. Diarrhœa, or great susceptibility of the bowels to purgatives is laid down as one of the characteristics of Typhoid Fever, and as a diagnostic symptom in the comparison with Typhus Fever. A case has recently occurred to the writer, of Typhoid Fever, lasting 3 or 4 weeks, in which there was *constipation*, only removed by good doses of purgatives, existing in a family where other cases were prevailing, one of them very severe, and well marked. There could be no mistake by any possibility concerning its true nature, all the other symptoms existing, but irritability of the bowels. Another case has recently occurred in the practice of a medical friend entirely similar. The tongue described as peculiar to this fever frequently does not exist, even in the severe cases—in place of being dry, and glazed, and red, we find it moist, and only slightly furred, and in place of sordes on the gums and teeth, we find them clean. The rose colored spots so often mentioned as very characteristic, and by some as almost pathognomonic, are absent in our latitude in many, probably much more than half the cases. Louis says, out of fifty-seven severe cases he examined, they were only absent in three, and not certainly in those, as the patients were not seen unti they may have faded. Chomel says, they are absent only in one fourth the number. Wood says they are very rarely absent. Such is not the experience of the Medical men of Virginia.

A variety of continued fever with two paroxysms in twenty-four hours has been noticed. Cullen, and probably Good, among the old writers allude to it—none of the moderns, as far as I have been able to ascertain, do. Dr. Wilson, late editor of the "*Stethescope*," in the November No., 1856, of that Journal, has written an article on this subject, and claims these two paroxysms as pathognomonic of *continued* fever. Dr. Mettauer, of Prince Edward, Va., in more hurry to claim the very early observation of this fact, than to prove its truth, comes to the same conclusion—that the two paroxysms in twenty-four hours, is Diagnostic of continued fever. His paper is published in the Stethescope (January, 1857.) We allude to this subject, as an evidence of this variety of continued fever, but with a firm conviction that it is not a frequent condition, and feel sure that the observation of almost every one who has treated continued fever, will confirm the statement of Watson, Wood, Louis, Bartlett, and others on this point, of one slight remission, and one paroxysm in twenty-four hours.

It is scarcely necessary to allude to the multiform varieties of Hysteria. Every Practitioner has been warned, and is on the look-out for the freaks of this protean malady. Still the inexperienced will sometimes be deceived, and possibly too the experienced will make a false diagnosis in some of the many, and wonderful forms Hysteria puts on.

Syphilis, is another disease of multiform appearance, though of late years, French observers have, we believe, properly made two classes of Syphilis, in which all the varieties may be included. If the Practitioner watches as his guide, for the chancre with indurated base, well defined edges, circular, and depressed in centre, as laid down by Hunter, he will doubt about many of his cases being Syphilis, or even venereal. If he adopts the division of Clerc into two kinds, one non-contaminating, with superficial, ill-defined chancre—with bubo ending in free suppuration, and the other variety with well defined, indurated chancre, and indurated bubo, rarely suppurating, contaminating in its character, he will be relieved of his doubts, and difficulties, and know where to place his cases.

We believe we have alluded to a sufficient number of diseases to answer our end—to prove to the Medical man that there is much variety in human maladies, and to induce him to practice close observation and experience in scanning "the ills flesh is heir to."

